# Label-Free Quantitative Proteomics in a Methylmalonyl-CoA Mutase-Silenced Neuroblastoma Cell Line

**DOI:** 10.3390/ijms19113580

**Published:** 2018-11-13

**Authors:** Michele Costanzo, Armando Cevenini, Emanuela Marchese, Esther Imperlini, Maddalena Raia, Luigi Del Vecchio, Marianna Caterino, Margherita Ruoppolo

**Affiliations:** 1 Dipartimento di Medicina Molecolare e Biotecnologie Mediche, Università degli Studi di Napoli “Federico II”, 80131 Naples, Italy; michele.costanzo@unina.it (M.C.); armando.cevenini@unina.it (A.C.); 2 CEINGE—Biotecnologie Avanzate s.c.ar.l., 80145 Naples, Italy; emanuela.marchese89@gmail.com (E.M.); raia@ceinge.unina.it (M.R.); luigi.delvecchio@unina.it (L.D.V.); 3 Associazione Culturale *DiSciMuS* RFC, Casoria, 80026 Naples, Italy; 4 Dipartimento di Salute Mentale e Fisica e Medicina Preventiva, Università degli Studi della Campania “L. Vanvitelli”, 80138 Naples, Italy; 5 IRCCS SDN, 80142 Naples, Italy; esther.imperlini@unina.it

**Keywords:** quantitative proteomics, Methylmalonic Acidemias (MMAs), Methylmalonyl-CoA Mutase (MUT), energy metabolism, mitochondrial proteins

## Abstract

Methylmalonic acidemias (MMAs) are inborn errors of metabolism due to the deficient activity of methylmalonyl-CoA mutase (MUT). MUT catalyzes the formation of succinyl-CoA from methylmalonyl-CoA, produced from propionyl-CoA catabolism and derived from odd chain fatty acids β-oxidation, cholesterol, and branched-chain amino acids degradation. Increased methylmalonyl-CoA levels allow for the presymptomatic diagnosis of the disease, even though no approved therapies exist. MMA patients show hyperammonemia, ketoacidosis, lethargy, respiratory distress, cognitive impairment, and hepatomegaly. The long-term consequences concern neurologic damage and terminal kidney failure, with little chance of survival. The cellular pathways affected by MUT deficiency were investigated using a quantitative proteomics approach on a cellular model of MUT knockdown. Currently, a consistent reduction of the MUT protein expression was obtained in the neuroblastoma cell line (SH-SY5Y) by using small-interfering RNA (siRNA) directed against an MUT transcript (MUT siRNA). The MUT absence did not affect the cell viability and apoptotic process in SH-SY5Y. In the present study, we evaluate and quantify the alterations in the protein expression profile as a consequence of MUT-silencing by a mass spectrometry-based label-free quantitative analysis, using two different quantitative strategies. Both quantitative methods allowed us to observe that the expression of the proteins involved in mitochondrial oxido-reductive homeostasis balance was affected by MUT deficiency. The alterated functional mitochondrial activity was observed in siRNA_MUT cells cultured with a propionate-supplemented medium. Finally, alterations in the levels of proteins involved in the metabolic pathways, like carbohydrate metabolism and lipid metabolism, were found.

## 1. Introduction

Hereditary methylmalonic acidemias (MMAs) are severe autosomal recessive inborn errors of intermediary metabolism caused by the deficient activity of methylmalonyl-CoA mutase (MUT) or defects in the synthesis of 5-deoxyadenosyl cobalamin, the active form of vitamin B12 and the essential cofactor of MUT. MUT converts methylmalonyl-CoA into succinyl-CoA, a Krebs cycle intermediate. Methylmalonyl-CoA is produced from the catabolism of propionyl-CoA, derived from the degradation of cholesterol, branched-chain amino acids (valine, isoleucine, methionine, and threonine), and odd chain fatty acids β-oxidation [1]. The defect in the MUT protein causes an increase in the level of methylmalonyl-CoA, which is then converted into methylmalonic acid (MMA). Hereditary MMAs are included in newborn screening panels in several countries [2,3,4,5], allowing for a presymptomatic diagnosis of the disease. To this aim, targeted mass spectrometry-based metabolomics is a powerful tool to profile amino acids and acylcarnitines in a quantitative manner [6,7,8].

Isolated MMAs are caused by a complete (mut^0^) or partial (mut^−^) loss of MUT activity [9]. At the moment, no approved therapies exist for isolated MMAs. Patients are treated with dietary protein restriction and cofactor supplementation. Despite these treatments, the mortality carries on being around 20%, and the disease progression is characterized by acute metabolic instability, especially for mut^0^ patients [10]. Methylmalonic acidemia patients show hyperammonemia, ketoacidosis, lethargy, respiratory distress, cognitive impairment, and hepatomegaly. The long-term consequences concern neurologic damage and terminal kidney failure, with little chance of survival. The metabolic instability, disability, and death rate are reduced as a result of liver and/or combined liver/kidney transplantation [11,12]. However, transplantation utility is limited by the low number of liver donors, significant surgery risks, and high procedural costs [13]. Thus, efforts are devoted to develop new therapies as an alternative to transplantation [14].

Despite the elevated levels of MMA, biological fluids are used as a hallmark of the pathology, and its accumulation may account for multisystemic pathological dysfunction (especially at a neuronal, hepatic, and renal level), the molecular mechanisms underlying the damage induced by MMA are not fully detailed. 

In order to investigate the cellular pathways altered downstream by MUT deficiency, we used small-interfering RNA to knockdown the MUT protein expression in a neuroblastoma cell line, (namely SH-SY5Y). Label-free quantitative proteomics [15,16] were used to identify the proteins whose levels were found to be deregulated after MUT enzyme knockdown. We found deregulation in the levels of mitochondrial proteins, such as electron transfer flavoprotein subunit alpha and 2-oxoglutarate carrier, crucially involved in the mitochondrial oxido-reductive homeostasis balance. In addition, we also observed significant differences in the level of proteins enrolled in the metabolic pathways, such as carbohydrate metabolism (Gamma-enolase and fructose-bisphosphate aldolase C) and lipid metabolism (sphingomyelin phosphodiesterase 4 and sulfatase-modifying factor 2). The cellular pathways altered upon the MUT enzyme reduction may represent putative therapeutic targets, which should be possibly taken into account for the design of new therapies to alleviate patients’ clinical manifestations.

## 2. Results and Discussion

### 2.1. MUT Silencing

**Figure 1:**
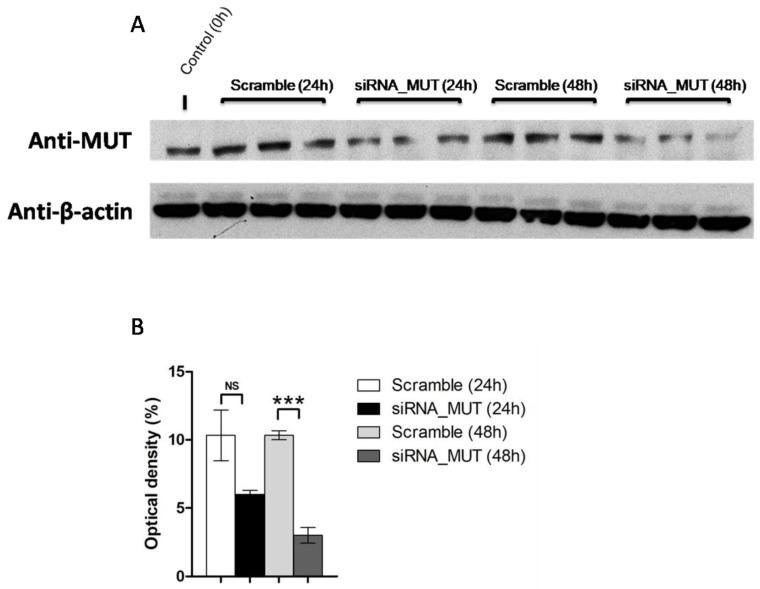
Reduction of methylmalonyl-CoA mutase (MUT) protein expression in SH-SY5Y cells. MUT silencing was evaluated 24 and 48 h after siRNA transfection by Western blot analysis using MUT specific antibodies. The silencing was carried out in three independent experiments at 24 and 48 h (**A**). The MUT optical density was measured and normalized by β-actin protein signal pixels (**B**). The results are reported as the mean ± standard deviation (SD). Statistical significance was calculated by one-way two tail paired *t*-test. *p*-values are indicated as follows: NS = non significant = *p* > 0.05; *** = *p* < 0.005.

The human SH-SY5Y cell line was transfected using a specific siRNA to reduce the expression of MUT protein (siRNA_MUT). A siRNA molecule unable to target known cellular transcripts was used as the negative control (Scramble). The silencing was evaluated 24 and 48 h after siRNA transfection. As shown in Figure 1, the MUT protein expression was reduced by about 50% after 24 h and by about 70% (*p* < 0.001) after 48 h. The 48-h time point was chosen for the following experiments.

### 2.2. Cell Survival and Apoptosis

**Figure 2:**
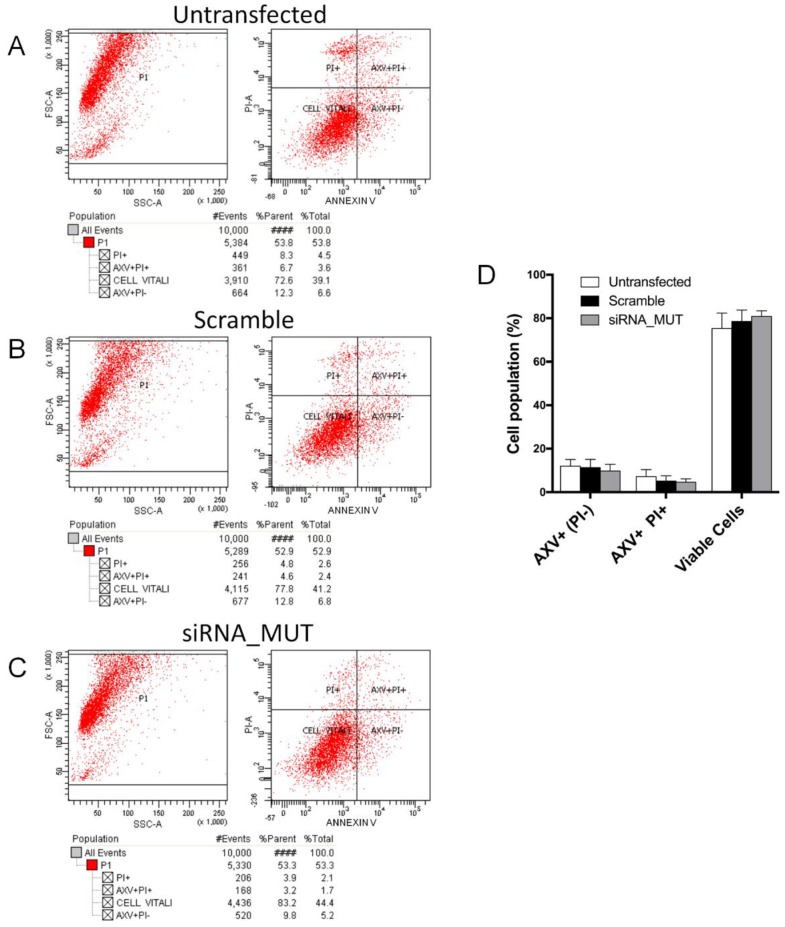
Analysis of apoptosis in siRNA_MUT cells. Apoptosis was assessed by Annexin V-FITC and PI staining and cytofluorimetric analysis in untrasfected (**A**) Scramble (**B**) and siRNA-MUT (**C**) cells. The percentage of cell populations are reported as the mean of three independent experiments ± SD (**D**). No significant difference was observed. AXV+, Annexin V positive cells; AXV+ PI+, Annexin V and propidium iodide positive cells.

The apoptosis rate was measured in MUT silenced SH-SY5Y (siRNA_MUT) 48 h after transient transfection. The cells transfected with scramble siRNA (Scramble) and untransfected cells have been used as the controls. The cells were stained with Annexin V and propidium iodide (PI), and analyzed by flow cytometry in order to evaluate the possible differences in the apoptotic rates. Indeed, the results revealed a very low percentage of cells with a high Annexin V signal and low PI signal (cells in early apoptosis), with no significant difference between the siRNA_MUT and Scramble cells (Figure 2). The percentage of healthy cells with both low (Annexin V and propidium iodide) signals was unaffected by the MUT silencing, as well as the percentage of cells with both high signals (representing cells in necrotic or late apoptotic state), which similarly showed no significant variation. A very low percentage of cells with high Annexin V and low PI signal (cells in early apoptosis) was present in all of the samples. This latter observation may indicate that the cells with both high signals were probably necrotic with the absence of apoptotic processes. In the examined temporal window, MUT silencing slightly affected cell viability without modifying the apoptotic rate, if compared with the Scramble siRNA transfection. In order to provide a quantitative estimation of the number of viable cells in the culture, a neutral-red uptake assay [17] was performed (Supplemental Figure S1) 48 h after transfection. Differences in the Scramble and siRNA_MUT cell viabilities were not observed. Moreover, the cell viability was comparable to the control untransfected cells.

### 2.3. Proteomic Profiles 

A quantitative proteomic analysis was performed using the human SH-SY5Y cell line, in which the MUT expression was reduced 48 h after transient transfection with siRNA against MUT. The cells transfected with scramble siRNA and harvested at the same time point (48 h) have been chosen the as proteomic experiment control.

**Figure 3:**
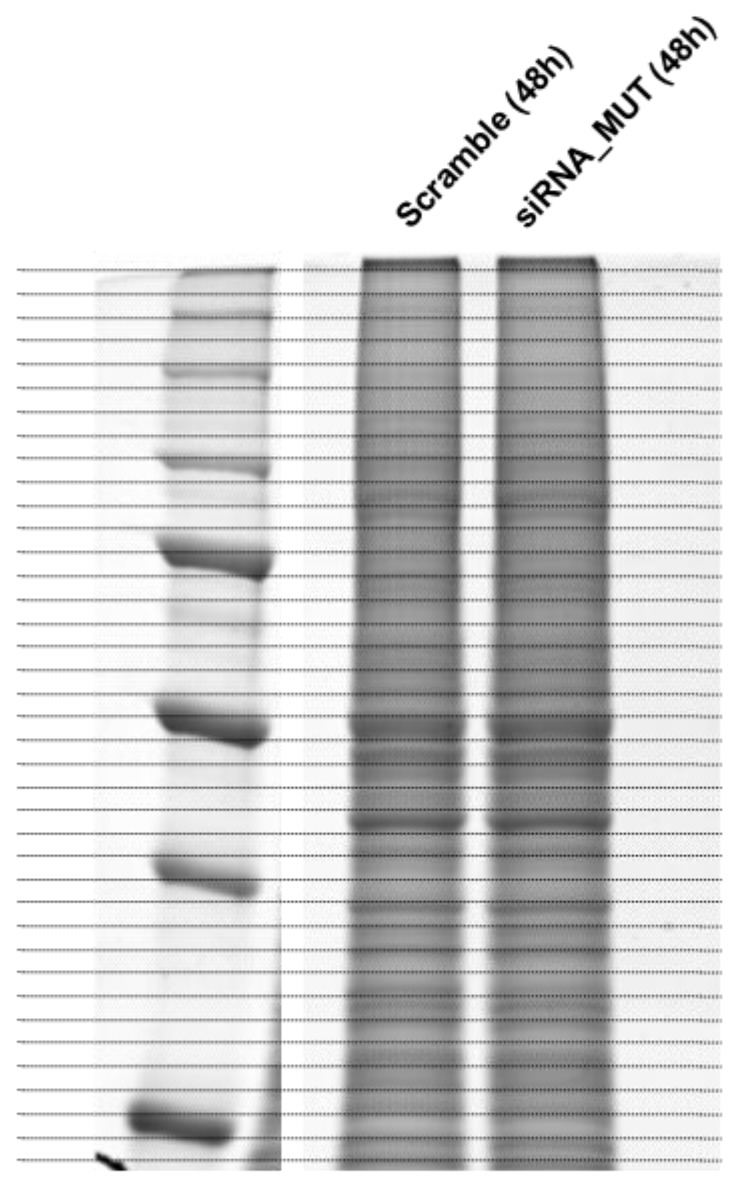
siRNA_MUT and scramble SH-SY5Y cell proteomes. Protein extracts were resolved on a 10% SDS-polyacrylamide gel and stained by a gel code blue stain reagent. Each gel lane was fractionated in order to obtain 40 fractions.

Cellular proteomes were resolved on a 10% Sodium Dodecyl Sulphate (SDS)-polyacrylamide gel (Figure 3). Each gel lane was fractionated in order to obtain 40 fractions, which were cut and properly processed for protein identification by nanoLC-MS/MS [18,19]. The protein species identified by more than three peptides were taken into account and included in our proteomic dataset. The resulted proteomic dataset was constituted by about 1000 proteins in both siRNA_MUT and Scramble cells. The details of the protein identification are reported in Supplemental Table S1. Label-free proteomic analysis was performed to estimate the relative abundance of each protein by two spectral counting parameters, R_SC_ and Fold_NSAF_ [20]. The spectral counting parameters, R_SC_ and Fold_NSAF_, were correlated by the Pearson correlation coefficient r = 0.9788, R^2^ = 0.9581, *p* < 0.0001 (Supplemental Figure S2 and Table S2).

According to the R_SC_ and Fold_NSAF_ values, the proteins were accepted to be deregulated when both of the following conditions occurred: R_SC_ > +3.5 or <−3.5; Fold_NSAF_ > +3.5 or <−3.5. R_SC_ and Fold_NSAF_ values referring to the differentially expressed proteins are reported in Table 1. The two different quantitative analytical indices identified 57 more abundant and 56 less abundant proteins in the silenced cells (siRNA_MUT).

### 2.4. Functional and Biological Annotation 

**Figure 4:**
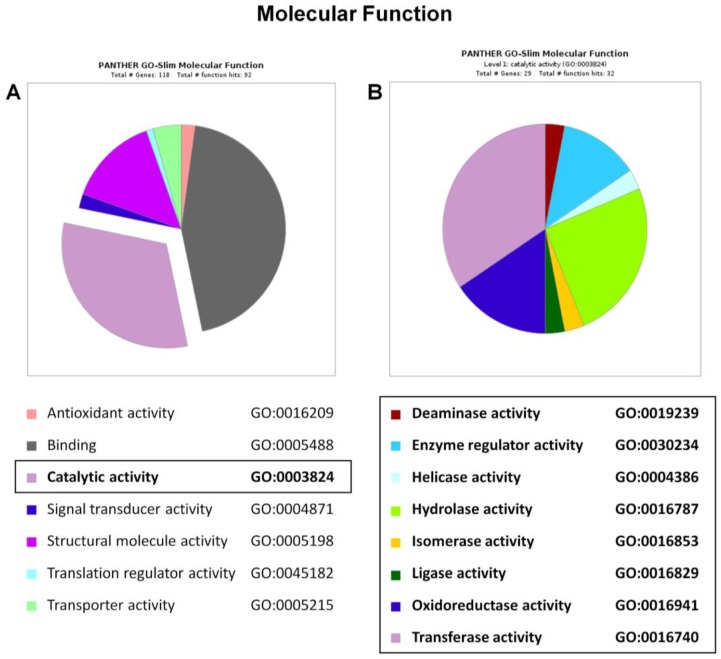
Molecular function classification of siRNA_MUT SH-SY5Y proteome. The differentially expressed proteins in siRNA_MUT SH-SY5Y cells versus control were clusterized according to their gene ontology molecular function using the Protein Analysis Through Evolutionary Relationship (PANTHER) software. The “catalytic activity” category is boxed (**A**) and detailed in panel (**B**).

In order to elucidate the implications of the differentially expressed proteins in the cellular processes subsequent to MUT silencing, we analyzed the identified proteins using the Protein Analysis Through Evolutionary Relationship (PANTHER) and Reactome databases. The PANTHER enrichment analysis allowed to cluster 92/118 deregulated hits according to their molecular function, as reported in Figure 4A. The following three main categories were identified: 41 hits as binding proteins (34.7–44.6%), 29 hits as proteins involved in catalytic activity (24.6–31.5%), and 13 hits as the proteins involved in the structural molecular activity (11.0–14.1%). We focused on the proteins involved in catalytic activity. This class was further subdivided into eight enzymatic categories, as reported in Figure 4B and Supplemental Table S3. Within the oxidoreductase activity category, the most interesting proteins are represented by electron transfer flavoprotein subunit alpha, mitochondrial (P13804, ETFA, Rsc = −3.68, and Fold_NSAF_ = −7.04), and Peroxiredoxin-6 (P30041, PRDX6, Rsc = −3.68, and Fold_NSAF_ = −3.73), both found to be less abundant in the siRNA_MUT sample. ETFA is a crucial enzyme involved in mitochondrial fatty acid β-oxidation, shuttling electrons from flavoprotein dehydrogenases, and the membrane-bound ubiquinone oxidoreductase. The impairment of ETFA-mediated processes affects the intracellular acidity. The type II glutaric aciduria is an example of the ETFA defect, characterized by glutaric, lactic, ethylmalonic, butyric, isobutyric, 2-methyl-butyric, and isovaleric acid accumulation. Moreover, PRDX6 is involved in the cell redox homeostasis, playing a protective role against oxidative stress. The PRDX6 down-regulation could affect the short chain fatty acid and phospholipid hydroperoxides reduction. Recently, it was reported that PRDX6 is involved in liver damage induced by oxidative stress [21]. Indeed, a deep linkage exists between methylmalonic acidemia and oxidative metabolism dysfunction [22,23,24]. Previous studies have already shown that increased MMA levels affect mitochondrial morphology and cytochrome c oxidase activity in patients [25,26].

**Figure 5:**
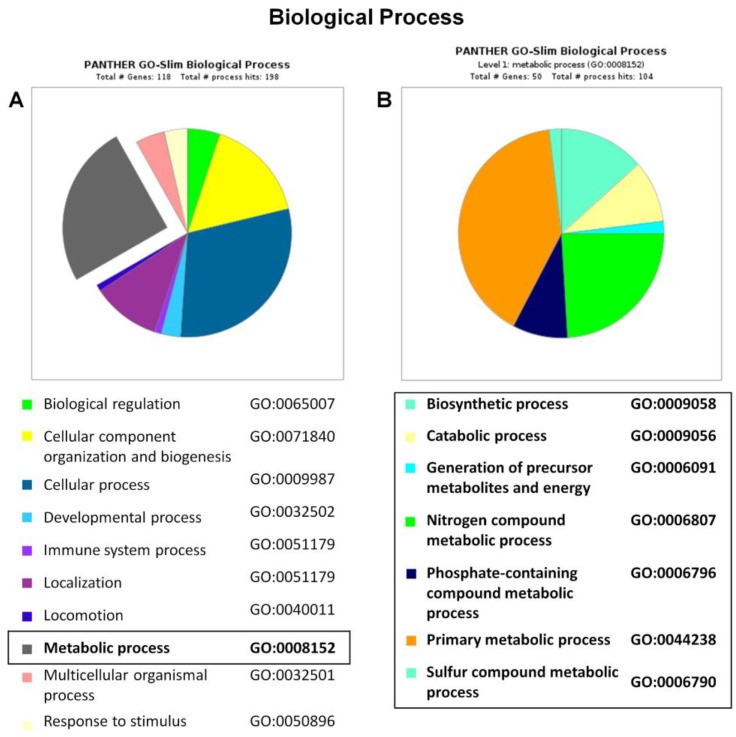
Biological process classification of siRNA_MUT SH-SY5Y proteome. The differentially expressed proteins in siRNA_MUT SH-SY5Y cells versus control were clusterized according to their gene ontology biological process using PANTHER software. The “metabolic process” category is boxed (**A**) and detailed in panel (**B**).

The PANTHER enrichment analysis was also performed to define the main biological processes involved in the identified protein dataset (Figure 5A). The following two main categories were identified: 59 hits as cellular process (29.8–50.0%) and 50 hits as metabolic process (25.3–42.4%). In the metabolic process, seven different subcategories were found and reported in Figure 5B and Supplemental Table S4. Gamma-enolase (P09104, ENOG, Rsc = −3.68, Fold_NSAF_ = −6.16) and fructose bisphosphate aldolase C (P09972, ALDOC, Rsc = −3.68, Fold_NSAF_ = −6.85), involved in energetic metabolism, were under-represented in the siRNA_MUT cells. In our previous proteomic investigation about the proteomic profiles of patients’ transplanted liver tissues [27], we also observed a decreased cellular level of proteins involved in the energy metabolism, gluconeogenesis, and Krebs cycle anaplerosis. The deregulation of the glucose metabolism, referred to “glycolysis” (*p*-value 1.31 × 10^−5^, False Discovery Rate (FDR) 4.46 × 10^−4^) and “gluconeogenesis” (*p*-value 6.57 × 10^−3^, FDR 1.31 × 10^−2^), was also confirmed by the Reactome database (Supplemental Table S5). In particular, M2OM belongs to the mitochondrial carrier protein family, controls the transport of 2-oxoglutarate across the inner mitochondrial membrane, and regulates the malate-aspartate and oxoglutarate-isocitrate shuttles. It also takes part in the nitrogen metabolism. Moreover, M2OM is responsible for glutathione uptake [28], and the glutathione deficiency is a complication of methylmalonic acidemia. Low levels of glutathione affect the cellular oxidative stress. As already mentioned above, evidences show once again a strong connection between methylmalonic acidemia and oxidative metabolism dysfunction [22,23,24]. These results are consistent with our previous studies showing disturbances in the glutathione metabolism in the lymphocytes of patients with cblC defect-associated MMAs [29].

Reactome also mapped five proteins involved in the “Cellular response to Hypoxia” (five hits; *p*-value 1.56 × 10^−3^, FDR 4.76 × 10^−3^) (Supplemental Table S5). Hypoxia has been recently associated with metabolic acidosis and with methylmalonic acidemia [30]. Moreover, the Reactome tool showed four hits for the involvement of proteins in the “lipid metabolism” (four hits; *p*-value 1.68 × 10^−1^, FDR 1.68 × 10^−1^) (Supplemental Table S5). Lipid metabolism involves SMPD4/NSMA3 and SUMF2, which belong to the glycosphingolipidic metabolism; SMPD4/NSMA3 is over-represented in the cells not-expressing MUT; it could be a protein of relevant interest, because it catalyzes the hydrolysis of membrane sphingomyelin to form phosphorylcholine and ceramide. A significant myelin content reduction was observed in the *cerebrum* from rat brains after the administration of MMA [31]. The MMA content may be related to the delayed myelination/cerebral atrophy and neurological dysfunction found in methylmalonic acidemia children. Conversely, the SUMF2 results are down-represented in cells not-expressing MUT; it heterodimerizes with another member of the same protein family, which is characterized by enzymatic activity that is able to generate C-alpha-formylglycine and activate sulfatases after the oxidation of cysteine residues to C-alpha-formylglycine. Multiple sulfatase deficiency (MSD; OMIM 272200) is a rare autosomal recessive inborn error of metabolism caused by mutations in the sulfatase modifying factor 1 gene, resulting in tissue accumulation of sulfatides, sulphated glycosaminoglycans, sphingolipids, and steroid sulfates [32].

### 2.5. MUT Silencing Decreases Cell Viability and Mitochondrial Functionality in Propionate-Enriched Culture Medium

**Figure 6:**
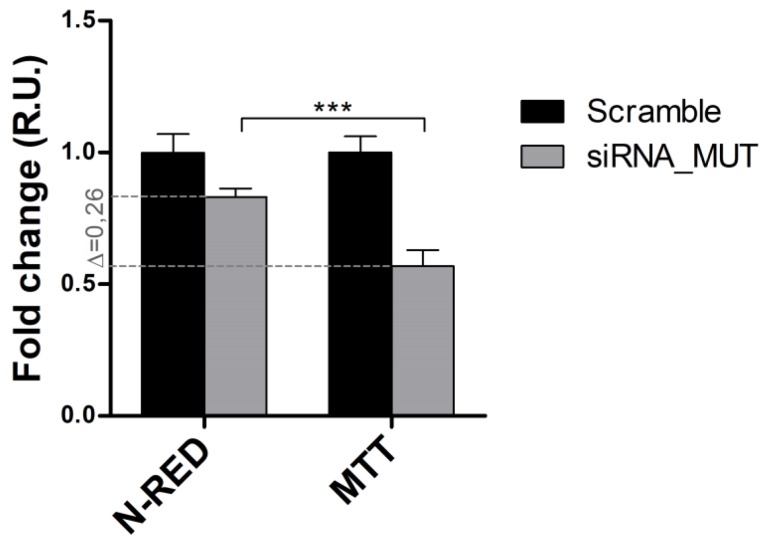
Cell viability and mitochondrial functionality assays of siRNA_MUT cells in sodium propionate-medium. Neutral-red and MTT assays were performed using 4.2 × 10^2^ cells/mm^2^, cultured in a medium containing 25 mM sodium propionate, 48 h after transfection. The Scramble and siRNA_MUT cells were compared. The signals were expressed as relative units (R.U.). The variation between the MTT and neutral-red fold-changes was reported (∆ = 0.26). Experiments were performed in three independent replicates. The results are reported as the mean ± SD. A one-way two-tail paired *t*-test was used to calculate the statistical significance (*p*-value); *** = *p* < 0.005.

Studies on cells carrying defects of MUT have been performed elsewhere after addition, in the culture medium, of metabolic precursors (e.g., propionate) of methylmalonil-CoA, in order to make more evident pathway unbalances [33]. A propionate-enriched culture medium was used to investigate whether the MUT knock-down could affect the cell viability and mitochondria functionality, using Scramble cells as the control. The siRNA_MUT cells cultured in a propionate-supplemented medium showed a slightly reduced cell viability, but a significant decreased mithocondrial functionality (Figure 6). The variation between the fold-changes of 3-(4,5-dimethylthiazol-2-yl)-2,5-diphenyltetrazolium bromide (MTT) and neutral-red assays was calculated as ∆ = 0.26. The reduced mitochondrial functionality, showed by a mitochondrial succinate dehydrogenase-based assay, supports the proteomic results showing the deregulation of a group of mitochondrial proteins.

## 3. Material and Methods

### 3.1. Cell Culture and Small Interfering RNA Transfection

SH-SH5Y cells (human bone marrow neuroblastoma; ATCC no. CRL-2266) were cultured in Dulbecco’s Modified Eagle Medium (DMEM) (Sigma-Aldrich, St. Louis, MO, USA) supplemented with 15% fetal bovine serum (FBS) (Gibco/Life Technologies, Rockville, MA, USA) and 2 mM l-Glutamine (EuroClone, Paington, UK). One day before transfection, 4.2 × 10^2^ cells/mm^2^ were seeded into 60 mm-diameter plates. The transfections were performed using 100 pmol/mL MUT siRNA (sc-95089, Santa Cruz Biotechnology, Dallas, TX, USA) and 0.05 μL Lipofectamine^®^ 2000Reagent (Thermo Fisher, Waltham, MA, USA) to pmol siRNA. After 5 h of incubation with Lipofectamine-siRNA complexes, the culture medium was replaced with fresh medium. The transfected cells were collected at two time points (24 and 48 h), while the untransfected cells were used as an additional control (0 h time point). Each experiment (time point) was performed in three independent replicates. The cells transfected with a negative control siRNA (scramble siRNA) (sc-37007, Santa Cruz Biotechnology, Dallas, TX, USA), following identical procedures adopted for MUT siRNA, were used as the control.

### 3.2. Cellular Lysis and Western Blotting Analysis

The cells were washed twice with an ice-cold PBS buffer and were mechanically removed from the plates by scraping in presence of a PBS buffer. They were centrifuged at 250 Relative Centrifugal Force (RCF) for 10 min at 4 °C. The supernatant of PBS was removed and the cellular pellets were incubated at 4 °C for 15 min in a lysis buffer containing 40 mM Tris pH = 8.6, 7 M urea, 2 M thiourea, 4% 3-[(3-cholamidopropyl)dimethylammonio]-1-propanesulfonate (CHAPS), and protease inhibitor cocktail (Roche, Indianapolis, IN, USA). The cellular lysates were sonicated and then centrifuged at 4 °C for 10 min at 15,000 RCF to remove the cellular debris. The supernatants were recovered and the protein concentration was measured using a 2D Quant kit (GE Healthcare, Piscataway, NJ, USA). The total protein extract (40 µg) were fractionated on 10% SDS-PAGE, transferred on a polyvinylidene fluoride (PVDF) membrane and analyzed by Western blot with a mouse monoclonal antibody anti-MUT (sc-136541, Santa Cruz Biotechnology, Dallas, TX, USA) at a 1:1000 dilution in 1% milk in 1X phosphate-buffered saline (PBS), 0.05% TWEEN-20 (Sigma Aldrich, St. Louis, MO, USA). Mouse monoclonal anti-β-actin (ab8226, Abcam, Cambridge, UK) was used as the internal control for immunoblotting at a dilution of 1:5000. Immunoblot detections were carried out using horseradish peroxidase-conjugated anti-mouse antibodies and enhanced chemiluminescence (GE Healthcare, Piscataway, NJ, USA). The signals were visualized by X-ray film exposure. The images were acquired by a GS-800 calibrated densitometer scan (Biorad, Hercules, CA, USA). The MUT protein pixels were quantified and normalized by β-actin protein signal pixels [34,35].

### 3.3. Apoptosis Assay by Flow Cytometry

The cells were transfected in 60 mm-diameter plates by using Scramble siRNA and MUT siRNA, respectively. After 48 h, the cells were detached from the plate by incubation for 2 min at 37 °C with Trypsin-EDTA (Sigma Aldrich, St. Louis, MO, USA), 1 mL of PBS was added to the plate, and the cellular suspension was recovered from the plate and centrifuged for 10 min at 250 RCF at 4 °C. The cell pellets were resuspended in 2 mL of ice-cold PBS and centrifuged again for 10 min at 250 RCF at 4 °C. The cell pellets were resuspended in 100 μL of 1X Binding Buffer (0.1 M HEPES pH = 7.4; 1.4 M NaCl; 25 mM CaCl_2_) containing 5 μL fluorescein isothiocyanate (FITC)-conjugated Annexin V (BD Biosciences, San Jose, CA, USA) and 5 μL propidium iodide (PI), for 15 min at room temperature in the dark. Subsequently, 400 μL of 1X Binding Buffer was added to each sample and the cells were analyzed, within 10 min using a FACSCanto II flow cytometer (BD Biosciences, San Jose, CA, USA). The cells with high Annexin V and low PI signals were considered to be in the early stages of the apoptotic process. The cells with high Annexin and PI signals were considered to be in late apoptosis or necrosis. The cell transfections and subsequent analyses were performed in three independent replicates.

### 3.4. Neutral-Red and MTT Assays

Both assays were performed as elsewhere reported [36]. Briefly, 48 h after propionate treatment (72 h after transfection), the cells were washed with PBS, and the culture medium was replaced with a fresh medium containing 0.5 mg/mL of MTT (Sigma-Aldrich, St. Louis, MO, USA) or 0.33 mg/mL neutral-red (Sigma-Aldrich, St. Louis, MO, USA). The cells were incubated for 2 h at 37 °C and then washed with PBS, which was completely removed. Then, for the MTT, a solution of 1 N hydrogen chloride–isopropanol (1:24, *v*:*v*) was pipetted to each well, and mixed to dissolve the dark-blue formazan crystals formed. After a few minutes of gentle agitation on a rocking platform at room temperature, the absorbance of each sample was read at 570 nm in a Perkin Elmer Enspire microplate reader. For the neutral-red assay, a solution of acetic acid–water–ethanol (1:49:49, *v*:*v*:*v*) was pipetted to each well to solubilize the dye, and after a few minutes of gentle agitation, the absorbance of each sample was read at 540 nm in the plate reader. For neutral-red and MTT assays, 4.2 × 10^2^ cells/mm^2^ were seeded into the wells of a 24-well microplate (Costar, Corning Inc., Corning, NY, USA). The transfections were performed as described above. 24 h after transfection, the culture medium was replaced with a fresh medium containing 25 mM sodium propionate. A neutral-red assay was performed onto untrasfected, Scramble, and siRNA_MUT cells. An MTT assay was performed onto Scramble and siRNA_MUT cells. The values were normalized versus untreated untransfected cells in the neutral-red assay and versus Scramble cells in the MTT assay, and expressed as relative units (R.U.). 

Experiments were performed in three independent replicates and the averages and standard deviations were reported on to the graphs. One-way two-tail paired *t*-test was used to calculate the statistical significance (*p*-value).

### 3.5. Proteomic Analysis

Aliquots (100 µg) of the protein extracts from three replicates for each cellular condition were fractionated on a preparative by 10% SDS-PAGE, 16 × 20 cm. The protein electrophoretic patterns were stained using Gel Code Blue Stain Reagent (Thermo Fisher Scientific, Waltham, MA, USA). Each gel lane was cut into 5 mm slices and these later were excised from gel. An in situ trypsin digestion of the slices was carried out [37,38,39,40]. Peptide mixtures were resuspended in 0.2% HCOOH and an MS analysis was performed using a LTQ-Orbitrap XL (Thermo Scientific, Bremen, Germany) coupled with nanoEASY II, Nanoseparations chromatographic system (75 µm–l 20 cm, column, Thermo Scientific, Bremen, Germany). The peptide mixture was concentrated and desalted onto a 2 cm trapping column (C18, ID 100 μm, 5 μm) and then fractionated onto 20 cm C18 reverse phase silica capillary column (ID 75 μm, 5 μm) (Nanoseparations). The peptides were eluted by a nonlinear gradient—4% B solvent (A eluent: 0.1% formic acid; B eluent: 80% acetonitrile, 0.08% formic acid) during 5 min, from 4 to 40% B in 45 min, and from 40 to 90% B in 1 min at flow rate of 250 nL/min [17]. An MS analysis was performed with a resolution set to 30000, and mass range from *m*/*z* 400 to 1800 Da. The three most intense doubly, triply, and fourthly charged ions were selected and fragmented using Collision Induced Dissociation (CID) fragmentation. A proteomic analysis was performed using a Proteome Discoverer™ platform (version 1.3.0.339; Thermo Scientific, Bremen, Germany), interfaced with an in-house Mascot server (version 2.3, Matrix Science, London, UK) for protein identifications. All of the peak lists were processed using the following parameters: (I) Spectrum Selector. Min Precursor Mass: 350 Da, Max. Precursor Mass: 5000 Da, Minimum Peak Count: 1; (II) Mascot: 1. Input Data. Protein Database: SwissProt, Enzyme: Trypsin, Maximum Missed Cleavage Sites: 2, Instrument: ESI-FTICR, Taxonomy: Homo sapiens. 2. Tolerances. Precursor Mass Tolerance: 5 ppm, Fragment Mass Tolerance: 0.8 Da. 3. Dynamic Modification. Methionine Oxidation, N-terminal Glutamine cyclization to Pyroglutamic Acid, N-terminal protein Acetylation. 4. Static modification. Cysteine Carboamidomethylation. Proteins identified by a minimum of two peptides were accepted [20].

### 3.6. Quantitative Label-Free Comparative Analysis

The spectral counting (SpC) approach [15,16] was used to compare the protein expression profiles of the siRNA_MUT cells with those of the negative control (Scamble). In order to perform a quantitative analysis, the abundances of the proteins present in each proteomes were estimated by means of the spectral counting, whereas the protein fold changes were expressed as R_SC_, calculated according to the following formula:

R_SC_ = log_2_ [(n2 + f)/ (n1 + f)] + log_2_ [(t1 – n1 + f)/(t2 − n2 + f)]

R_SC_ is the log ratio of abundance between samples 1 (Scramble) and 2 (siRNA_MUT); n1 and n2 are the SpCs for the given protein in sample groups 1 and 2, respectively; t1 and t2 are the total numbers of spectra over all of the proteins in the two sample groups; f is a correction factor set to 0.5 and used to eliminate discontinuity due to SpC = 0 [20]. The normalized spectral abundance factor (NSAF) for a given protein was calculated as the ratio of its spectral abundance factor SAF (SpC divided by protein length) and the sum of all SAFs for the proteins identified within that run. The NSAF values allow for comparing the relative abundance of proteins both between and within the samples. To measure the relative abundance for each protein identified in the dataset, Fold_NSAF_ was calculated as log_2_ (NSAF1/NSAF2), where NSAF1 is referred to siRNA_MUT, and NSAF2 to the Scramble conditions, respectively. Within the obtained datasets, proteins showing R_SC_ > +3.5 or R_SC_ <−3.5 and Fold_NSAF_ > +3.5 or <−3.5, were considered as differentially expressed between the analyzed groups. A statistical analysis was performed using the GraphPad Prism Version 5.03 (La Jolla, CA, USA). The R_SC_ and Fold_NSAF_ correlation was evaluated using the Pearson’s coefficient test.

### 3.7. Bioinformatic Analysis

To investigate the potential cellular processes affected by the MUT knockdown, we analyzed the identified proteomic dataset using PANTHER (Protein Analysis Through Evolutionary Relationship) database (Available online: http://www.pantherdb.org) [41,42]. Moreover, the Reactome database (Available online: https://www.reactome.org), an open-source and open access pathway database summarizing diverse pathway model collection, was also used to support the enrichment analysis and combine the pathway analysis with a functional classification of the differentially expressed proteins [43,44,45].

## 4. Conclusions

In summary, the proteomic characterization of a methylmalonyl-CoA mutase-silenced neuroblastoma cell line allowed us to define a dataset of deregulated proteins and relative alterated cellular pathways that may be investigated to highlight the unknown molecular mechanism underlying MMA damage. The identification of deregulated mitochondrial proteins is a key result of the definition of the molecular mechanisms involved in MMA pathophysiology. In fact, although it is clear that the increased methylmalonic acid levels affect the progression of the disease, the role of the mitochondria in this process has not been detailed yet.

## Figures and Tables

**Table 1:** Protein abundance based on R_SC_ and Fold_NSAF_ in siRNA_MUT cells.

Swiss-Prot Accession	Gene Name	Protein Descriptions	R_SC_	Fold_NSAF_
Q9Y678	*COPG1*	Coatomer subunit gamma-1	−4.62	−6.22
O14776	*TCRG1*	Transcription elongation regulator 1	−4.62	−5.89
Q02952	*TCRG1*	A-kinase anchor protein 12	−4.50	−5.07
P08865	*RSSA*	40S ribosomal protein SA	−4.37	−7.53
Q15365	*PCBP1*	Poly(rC)-binding protein 1	−4.37	−7.25
Q02978	*M2OM*	Mitochondrial 2-oxoglutarate/malate carrier protein	−4.37	−7.44
P11717	*MPRI*	Cation-independent mannose-6-phosphate receptor	−4.37	−6.89
P62913	*RL11*	60S ribosomal protein L11	−4.23	−4.45
P48643	*TCPE*	T-complex protein 1 subunit epsilon	−4.23	−8.10
O43242	*PSMD3*	26S proteasome non-ATPase regulatory subunit 3	−4.23	−6.50
Q15477	*SKIV2*	Helicase SKI2W	−4.23	−6.52
P50395	*GDIB*	Rab guanosine diphosphate dissociation inhibitor beta	−4.06	−5.30
Q14240	*IF4A2*	Eukaryotic initiation factor 4A-II	−4.06	−6.61
Q01433	*AMPD2*	AMP deaminase 2	−4.06	−6.74
P35222	*CTNB1*	Catenin beta-1	−4.06	−5.63
P04792	*HSPB1*	Heat shock protein beta-1	−3.88	−5.80
P00568	*KAD1*	Adenylate kinase isoenzyme 1	−3.88	−7.54
Q14232	*EI2BA*	Translation initiation factor eIF-2B subunit alpha	−3.88	−7.62
P09960	*LKHA4*	Leukotriene A-4 hydrolase	−3.88	−6.96
P43686	*PRS6B*	26S protease regulatory subunit 6B	−3.88	−5.96
Q14155	*ARHG7*	Rho guanine nucleotide exchange factor 7	−3.88	−6.51
Q13045	*FLII*	Protein flightless-1 homolog	−3.88	−5.57
Q14444	*CAPR1*	Caprin-1	−3.88	−6.29
P15924	*DESP*	Desmoplakin	−3.88	−6.56
Q96JQ0	*PCD16*	Protocadherin-16	−3.88	−5.92
Q9NR31	*SAR1A*	guanosine triphosphate (GTP)-binding protein SAR1a	−3.68	−4.91
Q9Y3B7	*RM11*	39S ribosomal protein L11, mitochondrial	−3.68	−5.75
P30041	*PRDX6*	Peroxiredoxin-6	−3.68	−3.73
P84103	*SRSF3*	Serine/arginine-rich splicing factor 3	−3.68	−3.53
P61020	*RAB5B*	Ras-related protein Rab-5B	−3.68	−7.36
Q13126	*MTAP*	*S*-methyl-5′-thioadenosine phosphorylase	−3.68	−7.41
P81605	*DCD*	Dermcidin	−3.68	−7.19
O00232	*PSD12*	26S proteasome non-ATPase regulatory subunit 12	−3.68	−7.64
Q9UFN0	*NPS3A*	Protein NipSnap homolog 3A	−3.68	−7.25
P09972	*ALDOC*	Fructose-bisphosphate aldolase C	−3.68	−6.85
Q8NBJ7	*SUMF2*	Sulfatase-modifying factor 2	−3.68	−8.21
P09104	*ENOG*	Gamma-enolase	−3.68	−6.16
P13804	*ETFA*	Electron transfer flavoprotein subunit alpha, mitochondrial	−3.68	−7.05
O15173	*PGRC2*	Membrane-associated progesterone receptor component 2	−3.68	−6.49
Q8NC51	*PAIRB*	Plasminogen activator inhibitor 1 RNA-binding protein	−3.68	−6.76
Q96HS1	*PGAM5*	Serine/threonine-protein phosphatase PGAM5, mitochondrial	−3.68	−6.23
Q9UJU6	*DBNL*	Drebrin-like protein	−3.68	−6.61
Q14194	*DPYL1*	Dihydropyrimidinase-related protein 1	−3.68	−7.19
Q96PZ0	*PUS7*	Pseudouridylate synthase 7 homolog	−3.68	−6.32
P62195	*PRS8*	26S protease regulatory subunit 8	−3.68	−6.82
Q14247	*SRC8*	Src substrate cortactin	−3.68	−6.25
Q9P289	*STK26*	Serine/threonine-protein kinase 26	−3.68	−5.83
Q9Y6E0	*STK24*	Serine/threonine-protein kinase 24	−3.68	−5.62
O14579	*COPE*	Coatomer subunit epsilon	−3.68	−6.33
Q13330	*MTA1*	Metastasis-associated protein MTA1	−3.68	−5.89
Q16401	*PSMD5*	26S proteasome non-ATPase regulatory subunit 5	−3.68	−6.29
Q15075	*EEA1*	Early endosome antigen 1	−3.68	−6.20
Q92626	*PXDN*	Peroxidasin homolog	−3.68	−6.73
O60841	*IF2P*	Eukaryotic translation initiation factor 5B	−3.68	−6.02
Q13576	*IQGA2*	Ras GTPase-activating-like protein IQGAP2	−3.68	−4.53
Q5VYK3	*ECM29*	Proteasome-associated protein ECM29 homolog	−3.68	−4.46
Q96GQ7	*DDX27*	Probable ATP-dependent RNA helicase DEAD box (DDX) 27	3.73	6.71
Q86VM9	*ZCH18*	Zinc finger (ZNF) CCCH domain-containing protein 18 O	3.73	6.46
Q9Y2A7	*NCKP1*	Nck-associated protein 1	3.73	5.89
Q9BUJ2	*HNRL1*	Heterogeneous nuclear ribonucleoprotein U-like protein 1	3.73	5.36
Q9Y6K1	*DNM3A*	DNA (cytosine-5)-methyltransferase 3A	3.73	5.39
Q9NXE4	*SMPD4*	Sphingomyelin phosphodiesterase 4	3.73	5.13
Q9BZJ0	*CRNL1*	Crooked neck-like protein 1	3.73	4.88
Q13523	*PRP4B*	Serine/threonine-protein kinase pre-mRNA-processing factor 4 homolog	3.73	5.28
Q8IXT5	*RB12B*	RNA-binding protein 12B	3.73	5.19
Q7KZ85	*SPT6H*	Transcription elongation factor suppressor of Ty6	3.73	5.33
O75691	*UTP20*	Small subunit processome component 20 homolog	3.73	5.29
P20340	*RAB6A*	Ras-related protein Rab-6A	3.93	5.05
Q13185	*CBX3*	Chromobox protein homolog 3	3.93	5.05
O14979	*HNRDL*	Heterogeneous nuclear ribonucleoprotein D-like	3.93	4.27
Q5BKZ1	*ZN326*	DBIRD complex subunit ZNF326	3.93	3.58
Q9Y3I0	*RTCB*	tRNA-splicing ligase RtcB homolog	3.93	7.54
Q96A65	*EXOC4*	Exocyst complex component 4	3.93	7.73
Q00325	*MPCP*	Phosphate carrier protein, mitochondrial	3.93	6.53
O60282	*KIF5C*	Kinesin heavy chain isoform 5C	3.93	6.06
Q68E01	*INT3*	Integrator complex subunit 3	3.93	6.26
Q13620	*CUL4B*	Cullin-4B	3.93	5.32
P51531	*SMCA2*	Probable global transcription activator SNF2L2	3.93	6.74
O00299	*CLIC1*	Chloride intracellular channel protein 1	4.11	5.34
P83916	*CBX1*	Chromobox protein homolog 1	4.11	5.22
Q96E39	*RMXL1*	RNA binding motif protein, X-linked-like-1	4.11	5.41
Q14978	*NOLC1*	Nucleolar and coiled-body phosphoprotein 1	4.11	4.61
Q15061	*WDR43*	WD repeat-containing protein 43	4.11	7.52
Q8WTT2	*NOC3L*	Nucleolar complex protein 3 homolog	4.11	7.91
P23921	*RIR1*	Ribonucleoside-diphosphate reductase large subunit	4.11	6.83
O75400	*PR40A*	Pre-mRNA-processing factor 40 homolog A	4.11	5.99
P52948	*NUP98*	Nuclear pore complex protein Nup98-Nup96	4.11	6.03
O75367	*H2AFY*	Core histone macro-H2A.1	4.27	5.79
O43290	*SNUT1*	U4/U6.U5 tri-snRNP-associated protein 1	4.27	5.81
O00571	*DDX3X*	ATP-dependent RNA helicase DDX3X	4.27	5.53
P39023	*RL3*	60S ribosomal protein L3	4.27	4.61
Q14690	*RRP5*	Protein RRP5 homolog	4.27	7.07
Q13151	*ROA0*	Heterogeneous nuclear ribonucleoprotein A0	4.42	5.96
P38919	*IF4A3*	Eukaryotic initiation factor 4A-III	4.42	6.24
Q9UMS6	*SYNP2*	Synaptopodin-2	4.42	6.95
Q9NYF8	*BCLF1*	Bcl-2-associated transcription factor 1	4.42	4.74
Q9H0A0	*NAT10*	*N*-acetyltransferase 10	4.42	7.51
Q9UKV3	*ACINU*	Apoptotic chromatin condensation inducer in the nucleus	4.42	7.08
P68431	*H31*	Histone H3.1	4.55	5.66
Q8IY81	*SPB1*	pre-rRNA processing protein FTSJ3	4.55	5.91
Q9H6R4	*NOL6*	Nucleolar protein 6	4.55	5.76
Q9NVP1	*DDX18*	ATP-dependent RNA helicase DDX18	4.55	5.37
Q8WUM0	*NU133*	Nuclear pore complex protein Nup133	4.67	8.81
Q8NI27	*THOC2*	THO complex subunit 2	4.67	6.17
P28331	*NDUS1*	Nicotinamide adenine dinucleotide-ubiquinone oxidoreductase 75 kDa subunit, mitochondrial	4.78	5.73
P07197	*NFM*	Neurofilament medium polypeptide	4.78	6.51
Q9UIG0	*BAZ1B*	Tyrosine-protein kinase bromodomain adjacent to ZNF 1B	4.78	5.85
P62805	*H4*	Histone H4	4.88	5.38
O00159	*MYO1C*	Unconventional myosin-Ic	4.98	6.63
Q9H583	*HEAT1*	HEAT repeat-containing protein 1	4.98	6.30
P28370	*SMCA1*	Probable global transcription activator SNF2L1	5.24	5.60
P49792	*RBP2*	E3 small ubiquitin-like modifier-protein ligase RanBP2	5.31	9.56
P0C0S5	*H2AZ*	Histone H2A.Z	5.38	6.29

## Supplementary Materials

The following are available online at http://www.mdpi.com/1422-0067/19/11/3580/s1, Supplemental Figure S1, Cell viability by Neutral-Red uptake assay; Supplemental Figure S2, Correlation between R_SC_ and Fold_NSAF_ parameters; Supplemental Table S1, Details of mass spectrometry identification; Supplemental Table S2, Complete list of protein abundances by R_SC_ and Fold_NSAF_ parameters; Supplemental Table S3, Catalytic activity classification by PANTHER database; Supplemental Table S4, Metabolic process classification by PANTHER database; Supplemental Table S5, Reactome database analysis.
