# Supplementary figures and images for: Label-Free Quantitative Proteomics in a Methylmalonyl-CoA Mutase-Silenced Neuroblastoma Cell Line
Source: Int J Mol Sci. 2018 Nov 13;19(11):3580. doi: 10.3390/ijms19113580 (PMC6275031; doi:10.3390/ijms19113580)

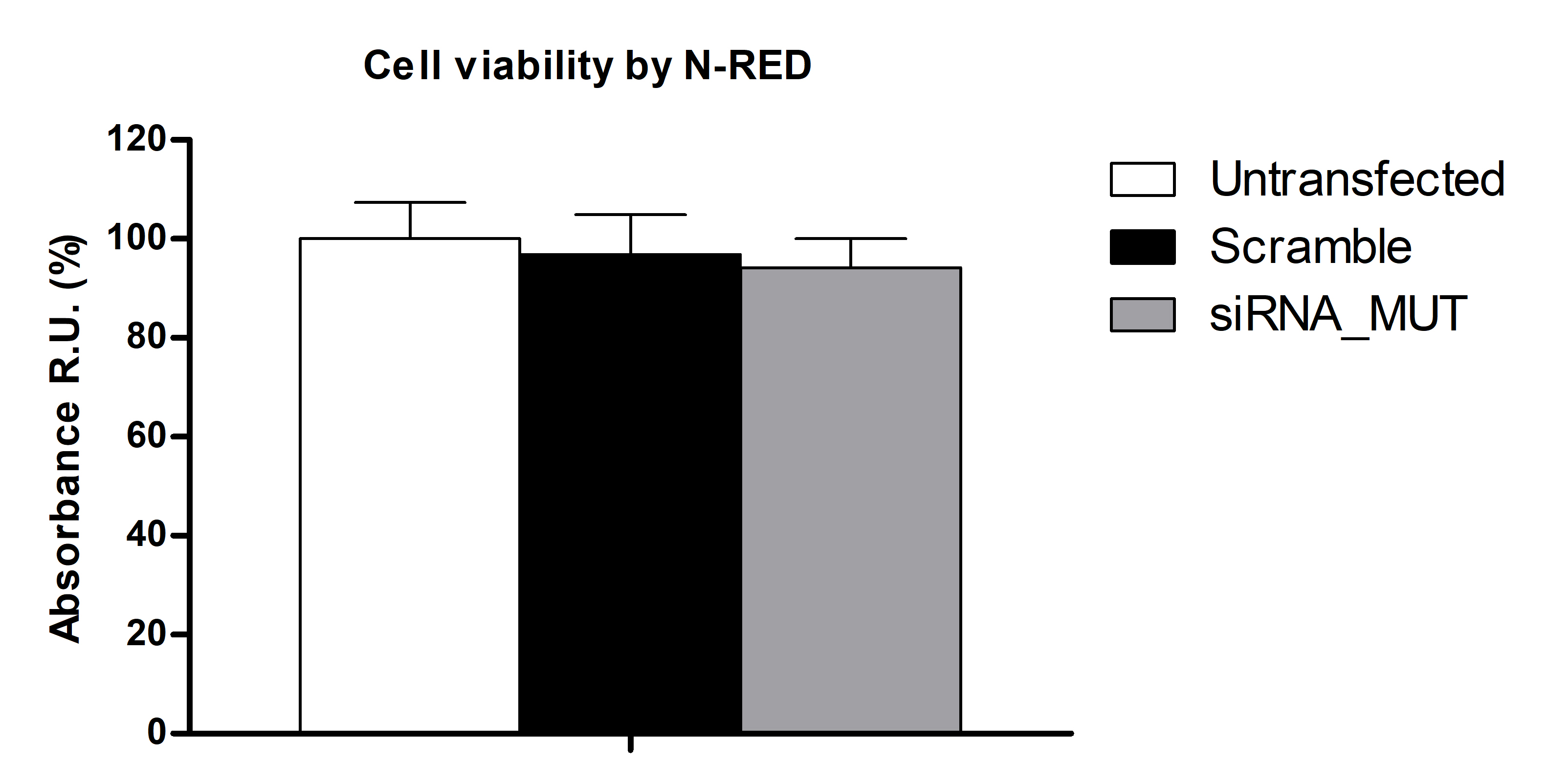

Supplement: Supplementary file 1 [file ijms-19-03580-s001.zip › ijms-389661 supplemtary/Supplemental Figure S1.jpg]

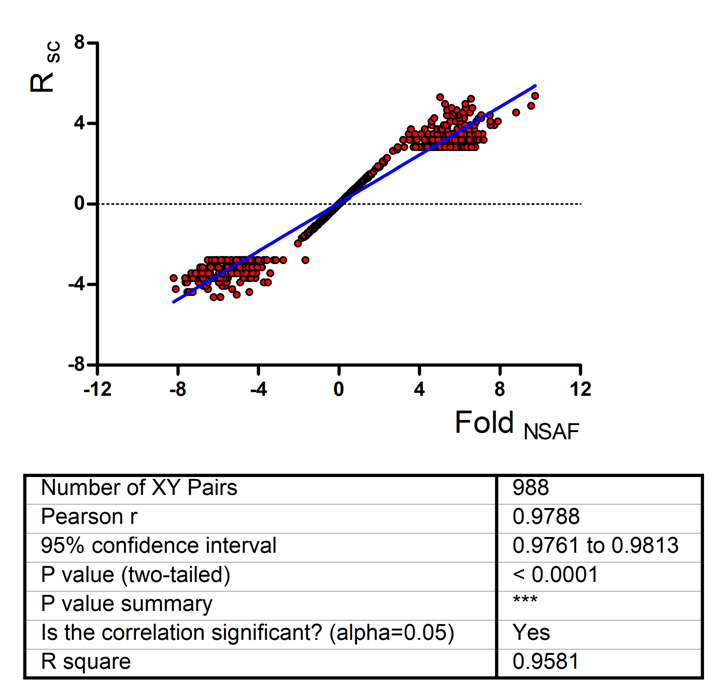

Supplement: Supplementary file 1 [file ijms-19-03580-s001.zip › ijms-389661 supplemtary/Supplemental Figure S2.png]
