# Supplementary material for: Label-Free Quantitative Proteomics in a Methylmalonyl-CoA Mutase-Silenced Neuroblastoma Cell Line
Source: Int J Mol Sci. 2018 Nov 13;19(11):3580. doi: 10.3390/ijms19113580 (PMC6275031; doi:10.3390/ijms19113580)
Supplement: Supplementary file 1 [file ijms-19-03580-s001.zip › ijms-389661 supplemtary/Supplemental Table S3 S4 S5.doc]

| **Supplemental Table S3. Catalytic activity by PANTHER Database** | |
| --- | --- |
| **Deaminase activity (GO:0019239)** | |
| Q01433 | AMP deaminase 2;AMPD2;ortholog |
| **Enzyme regulator activity (GO:0030234**) | |
| P49792 | E3 SUMO-protein ligase RanBP2;RANBP2;ortholog |
| O00159 | Unconventional myosin-Ic;MYO1C;ortholog |
| Q13576 | Ras GTPase-activating-like protein IQGAP2;IQGAP2;ortholog |
| P50395 | Rab GDP dissociation inhibitor beta;GDI2;ortholog |
| **Helicase activity (GO:0004386)** | |
| Q15477 | Helicase SKI2W;SKIV2L;ortholog |
| **Hydrolase activity (GO:0016787)** | |
| Q96PZ0 | Pseudouridylate synthase 7 homolog;PUS7;ortholog |
| P00568 | Adenylate kinase isoenzyme 1;AK1;ortholog |
| Q9H583 | HEAT repeat-containing protein 1;HEATR1;ortholog |
| Q8IXT5 | RNA-binding protein 12B;RBM12B;ortholog |
| Q8WUM0 | Nuclear pore complex protein Nup133;NUP133;ortholog |
| Q8WTT2 | Nucleolar complex protein 3 homolog;NOC3L;ortholog |
| P11717 | Cation-independent mannose-6-phosphate receptor;IGF2R;ortholog |
| Q9NY61 | Protein AATF;AATF;ortholog |
| **Isomerase activity (GO:0016853)** | |
| Q96PZ0 | Pseudouridylate synthase 7 homolog;PUS7;ortholog |
| **Lyase activity (GO:0016829)** | |
| P09104 | Gamma-enolase;ENO2;ortholog |
| **Oxidoreductase activity (GO:0016491)** | |
| P23921 | Ribonucleoside-diphosphate reductase large subunit;RRM1;ortholog |
| P13804 | Electron transfer flavoprotein subunit alpha, mitochondrial;ETFA;ortholog |
| Q92626 | Peroxidasin homolog;PXDN;ortholog |
| P28331 | NADH-ubiquinone oxidoreductase 75 kDa subunit, mitochondrial;NDUFS1;ortholog |
| P30041 | Peroxiredoxin-6;PRDX6;ortholog |
| **Transferase activity (GO:0016740)** | |
| P00568 | Adenylate kinase isoenzyme 1;AK1;ortholog |
| Q5VYK3 | Proteasome-associated protein ECM29 homolog;ECM29;ortholog |
| Q8IY81 | pre-rRNA processing protein FTSJ3;FTSJ3;ortholog |
| P49792 | E3 SUMO-protein ligase RanBP2;RANBP2;ortholog |
| Q9P289 | Serine/threonine-protein kinase 26;STK26;ortholog |
| Q9H0A0 | RNA cytidine acetyltransferase;NAT10;ortholog |
| Q13126 | S-methyl-5'-thioadenosine phosphorylase;MTAP;ortholog |
| O00299 | Chloride intracellular channel protein 1;CLIC1;ortholog |
| Q9Y6E0 | Serine/threonine-protein kinase 24;STK24;ortholog |
| P50395 | Rab GDP dissociation inhibitor beta;GDI2;ortholog |
| Q13523 | Serine/threonine-protein kinase PRP4 homolog;PRPF4B;ortholog |

| **Supplemental Table S4. Metabolic process by PANTHER Database** | |
| --- | --- |
| **Biosynthetic process (GO:0009058)** | |
| Q9H583 | HEAT repeat-containing protein 1;HEATR1;ortholog |
| Q8WUM0 | Nuclear pore complex protein Nup133;NUP133;ortholog |
| P23921 | Ribonucleoside-diphosphate reductase large subunit;RRM1;ortholog |
| P49792 | E3 SUMO-protein ligase RanBP2;RANBP2;ortholog |
| O75367 | Core histone macro-H2A.1;H2AFY;ortholog |
| P35222 | Catenin beta-1;CTNNB1;ortholog |
| P0C0S5 | Histone H2A.Z;H2AFZ;ortholog |
| P08865 | 40S ribosomal protein SA;RPSA;ortholog |
| Q13126 | S-methyl-5'-thioadenosine phosphorylase;MTAP;ortholog |
| O60841 | Eukaryotic translation initiation factor 5B;EIF5B;ortholog |
| Q7KZ85 | Transcription elongation factor SPT6;SUPT6H;ortholog |
| P28331 | NADH-ubiquinone oxidoreductase 75 kDa subunit, mitochondrial;NDUFS1;ortholog |
| O14776 | Transcription elongation regulator 1;TCERG1;ortholog |
| P05388 | 60S acidic ribosomal protein P0;RPLP0;ortholog |
| **Catabolic process (GO:0009056)** | |
| P13804 | Electron transfer flavoprotein subunit alpha, mitochondrial;ETFA;ortholog |
| P49792 | E3 SUMO-protein ligase RanBP2;RANBP2;ortholog |
| O00232 | 26S proteasome non-ATPase regulatory subunit 12;PSMD12;ortholog |
| P20340 | Ras-related protein Rab-6A;RAB6A;ortholog |
| O43242 | 26S proteasome non-ATPase regulatory subunit 3;PSMD3;ortholog |
| P30041 | Peroxiredoxin-6;PRDX6;ortholog |
| O60282 | Kinesin heavy chain isoform 5C;KIF5C;ortholog |
| Q15477 | Helicase SKI2W;SKIV2L;ortholog |
| Q13620 | Cullin-4B;CUL4B;ortholog |
| P61020 | Ras-related protein Rab-5B;RAB5B;ortholog |
| **Generation of precursor metabolites and energy (GO:0006091)** | |
| P09104 | Gamma-enolase;ENO2;ortholog |
| P09972 | Fructose bisphosphate aldolase C |
| P28331 | NADH-ubiquinone oxidoreductase 75 kDa subunit, mitochondrial;NDUFS1;ortholog |
| **Nitrogen compound metabolic process (GO:0006807)** | |
| Q96PZ0 | Pseudouridylate synthase 7 homolog;PUS7;ortholog |
| Q9H583 | HEAT repeat-containing protein 1;HEATR1;ortholog |
| Q8WUM0 | Nuclear pore complex protein Nup133;NUP133;ortholog |
| Q9H6R4 | Nucleolar protein 6;NOL6;ortholog |
| P23921 | Ribonucleoside-diphosphate reductase large subunit;RRM1;ortholog |
| Q8IY81 | pre-rRNA processing protein FTSJ3;FTSJ3;ortholog |
| P49792 | E3 SUMO-protein ligase RanBP2;RANBP2;ortholog |
| O75367 | Core histone macro-H2A.1;H2AFY;ortholog |
| P35222 | Catenin beta-1;CTNNB1;ortholog |
| P20340 | Ras-related protein Rab-6A;RAB6A;ortholog |
| Q9H0A0 | RNA cytidine acetyltransferase;NAT10;ortholog |
| P0C0S5 | Histone H2A.Z;H2AFZ;ortholog |
| P08865 | 40S ribosomal protein SA;RPSA;ortholog |
| Q13126 | S-methyl-5'-thioadenosine phosphorylase;MTAP;ortholog |
| O43290 | U4/U6.U5 tri-snRNP-associated protein 1;SART1;ortholog |
| Q7KZ85 | Transcription elongation factor SPT6;SUPT6H;ortholog |
| O00299 | Chloride intracellular channel protein 1;CLIC1;ortholog |
| P68431 | Histone H3.1;HIST1H3A;ortholog |
| O14776 | Transcription elongation regulator 1;TCERG1;ortholog |
| O75400 | Pre-mRNA-processing factor 40 homolog A;PRPF40A;ortholog |
| O60282 | Kinesin heavy chain isoform 5C;KIF5C;ortholog |
| Q15477 | Helicase SKI2W;SKIV2L;ortholog |
| Q12788 | Transducin beta-like protein 3;TBL3;ortholog |
| P61020 | Ras-related protein Rab-5B;RAB5B;ortholog |
| **Phosphate-containing compound metabolic process (GO:0006796)** | |
| P23921 | Ribonucleoside-diphosphate reductase large subunit;RRM1;ortholog |
| P49792 | E3 SUMO-protein ligase RanBP2;RANBP2;ortholog |
| P20340 | Ras-related protein Rab-6A;RAB6A;ortholog |
| Q9P289 | Serine/threonine-protein kinase 26;STK26;ortholog |
| Q9Y6E0 | Serine/threonine-protein kinase 24;STK24;ortholog |
| Q13523 | Serine/threonine-protein kinase PRP4 homolog;PRPF4B;ortholog |
| O60282 | Kinesin heavy chain isoform 5C;KIF5C;ortholog |
| Q15477 | Helicase SKI2W;SKIV2L;ortholog |
| P61020 | Ras-related protein Rab-5B;RAB5B;ortholog |
| P23921 | Ribonucleoside-diphosphate reductase large subunit;RRM1;ortholog |
| P49792 | E3 SUMO-protein ligase RanBP2;RANBP2;ortholog |
| P20340 | Ras-related protein Rab-6A;RAB6A;ortholog |
| **Primary metabolic process (GO:0044238)** | |
| Q96PZ0 | Pseudouridylate synthase 7 homolog;PUS7;ortholog |
| P00568 | Adenylate kinase isoenzyme 1;AK1;ortholog |
| Q9H583 | HEAT repeat-containing protein 1;HEATR1;ortholog |
| Q8IXT5 | RNA-binding protein 12B;RBM12B;ortholog |
| Q8WUM0 | Nuclear pore complex protein Nup133;NUP133;ortholog |
| Q9H6R4 | Nucleolar protein 6;NOL6;ortholog |
| Q5VYK3 | Proteasome-associated protein ECM29 homolog;ECM29;ortholog |
| P23921 | Ribonucleoside-diphosphate reductase large subunit;RRM1;ortholog |
| P13804 | Electron transfer flavoprotein subunit alpha, mitochondrial;ETFA;ortholog |
| Q01433 | AMP deaminase 2;AMPD2;ortholog |
| Q8IY81 | pre-rRNA processing protein FTSJ3;FTSJ3;ortholog |
| P49792 | E3 SUMO-protein ligase RanBP2;RANBP2;ortholog |
| O00232 | 26S proteasome non-ATPase regulatory subunit 12;PSMD12;ortholog |
| O75367 | Core histone macro-H2A.1;H2AFY;ortholog |
| P35222 | Catenin beta-1;CTNNB1;ortholog |
| P20340 | Ras-related protein Rab-6A;RAB6A;ortholog |
| P84103 | Serine/arginine-rich splicing factor 3;SRSF3;ortholog |
| Q9NYF8 | Bcl-2-associated transcription factor 1;BCLAF1;ortholog |
| Q9H0A0 | RNA cytidine acetyltransferase;NAT10;ortholog |
| P0C0S5 | Histone H2A.Z;H2AFZ;ortholog |
| P08865 | 40S ribosomal protein SA;RPSA;ortholog |
| Q13126 | S-methyl-5'-thioadenosine phosphorylase;MTAP;ortholog |
| O43290 | U4/U6.U5 tri-snRNP-associated protein 1;SART1;ortholog |
| Q7KZ85 | Transcription elongation factor SPT6;SUPT6H;ortholog |
| O43242 | 26S proteasome non-ATPase regulatory subunit 3;PSMD3;ortholog |
| O00299 | Chloride intracellular channel protein 1;CLIC1;ortholog |
| P68431 | Histone H3.1;HIST1H3A;ortholog |
| Q13330 | Metastasis-associated protein MTA1;MTA1;ortholog |
| Q96E39 | RNA binding motif protein, X-linked-like-1;RBMXL1;ortholog |
| Q15365 | Poly(rC)-binding protein 1;PCBP1;ortholog |
| P48643 | T-complex protein 1 subunit epsilon;CCT5;ortholog |
| Q14247 | Src substrate cortactin;CTTN;ortholog |
| O75400 | Pre-mRNA-processing factor 40 homolog A;PRPF40A;ortholog |
| O60282 | Kinesin heavy chain isoform 5C;KIF5C;ortholog |
| Q15477 | Helicase SKI2W;SKIV2L;ortholog |
| P05388 | 60S acidic ribosomal protein P0;RPLP0;ortholog |
| Q13620 | Cullin-4B;CUL4B;ortholog |
| Q12788 | Transducin beta-like protein 3;TBL3;ortholog |
| Q8NC51 | Plasminogen activator inhibitor 1 RNA-binding protein;SERBP1;ortholog |
| P61020 | Ras-related protein Rab-5B;RAB5B;ortholog |
| Q9BZJ0 | Crooked neck-like protein 1;CRNKL1;ortholog |
| Q14194 | Dihydropyrimidinase-related protein 1;CRMP1;ortholog |
| **Sulfur compound metabolic process (GO:0006790)** | |
| Q13126 | S-methyl-5'-thioadenosine phosphorylase;MTAP;ortholog |
| O00299 | Chloride intracellular channel protein 1;CLIC1;ortholog |

| **Supplemental Table S5. Reactome Database analysis** | | | | |
| --- | --- | --- | --- | --- |
| **Glycolysis** | | | | |
| P49792 | RBP2 | E3 SUMO-protein ligase Ran BP2 | 5,31 | 9,56 |
| Q8WUM0 | NU133 | Nuclear pore complex protein Nup 133 | 4,67 | 8,81 |
| P52948 | NUP98 | Nuclear pore complex protein Nup98-Nup96 | 4,11 | 6,03 |
| P09972 | ALDOC | Fructose-bisphosphate aldolase C | -3,68 | -6,85 |
| P09104 | ENOG | Gamma-Enolase | -3,68 | -6,16 |
| Q02978 | M2OM | Mitochondrial 2-oxoglutarate/malate carrier protein | -4,37 | -7,44 |
| **Gluconeogenesis** | | | | |
| P09972 | ALDOC | Fructose-bisphosphate aldolase C | -3,68 | -6,85 |
| P09104 | ENOG | Gamma-Enolase | -3,68 | -6,16 |
| Q02978 | M2OM | Mitochondrial 2-oxoglutarate/malate carrier protein | -4,37 | -7,44 |
| **Cellular response to Hypoxia** | | | | |
| Q16401 | PSMD5 | 26S proteasome non-ATPase regulatory subunit 5 | -3,68 | -6,29 |
| O43242 | PSMD3 | 26S proteasome non-ATPase regulatory subunit 3 | -4,23 | -6,5 |
| P62195 | PRS8 | 26S protease regulatory subunit 8 | -3,68 | -6,8 |
| O00232 | PSD12 | 26S proteasome non-ATPase regulatory subunit 12 | -3,68 | -7,64 |
| P43686 | PRS6B | 26S protease regulatory subunit 6B | -3,88 | -5,96 |
| **Lipid Metabolism** | | | | |
| Q9NXE4 | SMPD4 | Sphingomyelin phosphodiesterase 4 | 3,73 | 5,13 |
| Q8NBJ7 | SUMF2 | Sulfatase-modifying factor 2 | -3,68 | -8,21 |
| P09960 | LKHA4 | Leukotriene A-4 hydrolase | -3,88 | -6,96 |
